# Extreme Value Theory in Application to Delivery Delays

**DOI:** 10.3390/e23070788

**Published:** 2021-06-22

**Authors:** Marcin Fałdziński, Magdalena Osińska, Wojciech Zalewski

**Affiliations:** 1 Department of Econometrics and Statistics, Nicolaus Copernicus University, Gagarina 11, 87-100 Toruń, Poland; marf@umk.pl; 2 Department of Economics, Nicolaus Copernicus University, Gagarina 11, 87-100 Toruń, Poland; 3 Department of Logistics, Nicolaus Copernicus University, Gagarina 11, 87-100 Toruń, Poland; w.zalewski@umk.pl

**Keywords:** rare events, information, intelligent transport system (ITS), Extreme Value Theory (EVT), return level

## Abstract

This paper uses the Extreme Value Theory (EVT) to model the rare events that appear as delivery delays in road transport. Transport delivery delays occur stochastically. Therefore, modeling such events should be done using appropriate tools due to the economic consequences of these extreme events. Additionally, we provide the estimates of the extremal index and the return level with the confidence interval to describe the clustering behavior of rare events in deliveries. The Generalized Extreme Value Distribution (GEV) parameters are estimated using the maximum likelihood method and the penalized maximum likelihood method for better small-sample properties. The findings demonstrate the advantages of EVT-based prediction and its readiness for application.

## 1. Introduction

The Extreme Value Theory (EVT) evaluates both the magnitude and frequency of rare events and supports long-term forecasting. Delivery delays in transportation occur stochastically and rarely. However, they always increase costs, decrease consumers’ satisfaction, and lower the confidence of subcontractors in the supply chain. Thus, this paper refers to information-based vehicle planning and route analysis in road freight transportation. Telematics systems track the vehicles and send data to integrated transport management systems (TMS). They help to anticipate extreme time delays when both the transport operator and subcontractors have delivered a fleet.

This study aims to model and forecast rare events that appear as delivery delays in road transport using the tools provided by the EVT. The detailed research questions refer to the following issues:

RQ1: Is the Generalized Extreme Values (GEV) distribution able to model delivery delays data properly?

RQ2: What are the implications of estimated return level and extremal index for delivery planning?

Events that are rare in frequency and massive in magnitude have become important due to their negative impact. The Extreme Value Theory constitutes a framework allowing one to describe and measure such events. The EVT has had a vast array of applications. It covers, among many others, environmental data [1,2,3,4,5,6,7,8,9], risk management [10,11,12,13,14,15,16,17,18], but also earthquake size distribution [19] and athletic records [20].

In the literature on logistics and supply chain management, the Extreme Value Theory is rarely applied. One of the reasons is that logistics means the right organization. Supply chain management provides techniques to provide adequate services at every stage of the supply chain, including delivery. The focus is on optimizing activities to ensure timeliness, the security of goods, and service quality throughout the supply chain. Although quality management is currently highly developed and carefully evaluated, the risk of delivery delays remains an essential factor for the entire supply chain’s instability.

In recent publications on transportation, one can find several contributions to the theory and practice of the EVT. Firstly, the extreme value theory has been widely used in the case of safety on road analysis. Zheng and Sayed [21] proposed using the extreme value theory approach to conduct traffic conflict-based before-after analysis. The capability of providing a confident estimation of extreme events by the EVT approach drives the before-after analysis to shift from normal traffic conflicts to more severe conflicts, which are relatively rare but have more in common with actual crashes. The generalized Pareto distributions of traffic conflicts with a time-to-collision (TTC) of less than 0.5 s were applied. The results show an approximately 34% reduction in total extreme-serious conflicts in the analyzed area. Orsini et al. [22] used the EVT to predict the annual number of crashes in motorway cross-sections. Two traditional approaches, block maxima and peak-over-threshold, were applied to estimate EVT parameters for each cross-section. The authors found that both methods produced reliable predictions of annual rear-end collisions. Secondly, travel delays were the subject of the EVT. Esfeh et al. [23] proposed a new class of extreme value distribution called compound generalized extreme value (CGEV) distribution for investigating the effects of monthly and seasonal variation on extreme travel delays in road networks. They assumed that the frequency and severity of extreme events are highly correlated to the variation in weather conditions. The change in driving behavior varies according to road/weather conditions and contributes to the monthly and seasonal variation in observed extreme travel times.

Moreover, time delays in road transportation are typically considered from the perspective of congestion in the urban area within a temporal and spatial characteristics framework [24,25], intelligent transport systems [26], motorway traffic control [27], and the deviation between the actual and estimated shipment demand of containers [28].

The paper allows filling the gap between the usefulness of the EVT and its application in delivery operations being a part of a supply chain.

The methodology is applied based on real-time delay data obtained from a large brewery company operating in a European trademark network, which contracted a road transport company to provide delivery services. The data comes from the integrated transport management system.

The remaining of the paper is as follows. Section 2 introduces the methodology based on the extreme value theory. Section 3 details the data characteristics and empirical results. Section 4 contains discussion, and Section 5 concludes.

## 2. Extreme Value Theory—A Methodological Background

When modeling the maxima of a random variable, extreme value theory serves the same role as the central limit theorem plays when modeling sums of random variables. In both cases, the theory indicates limiting distributions. The Fisher and Tippett [29] theorem states that rescaled sample maxima converge in distribution to a variable having a distribution of Generalized Extreme Value (GEV) distribution:

(1) $$H_{\gamma ,\mu ,\sigma }\left(z\right)=\{\begin{array}{l} exp\left\{-{\left(1+\gamma \frac{z-\mu }{\sigma }\right)}^{-1/\gamma }\right\}\;\gamma \neq 0\\ exp\left\{-exp\left(-\left(\frac{z-\mu }{\sigma }\right)\right)\right\}\;\gamma =0 \end{array}$$

where 1+γ(z−μ)/σ>0, and −∞<μ<∞, σ>0 and −∞<μ<∞.

The most common approach for describing extreme events for stationary data is the block maximum method, which models the maxima of a set of contiguous blocks of observations using GEV distribution. The blocks method assumes a given sample, $X_{1},\ldots ,X_{N}$, divided into m blocks of length n, where the whole sample is N=nm. Then, the *j*-th block comprises $X_{\left(j-1\right)n+1},\ldots ,X_{jn}$. For each block, the maximum $M_{n,j}=max\left(X_{\left(j-1\right)n+1},\ldots ,X_{jn}\right)$ is calculated. The next step is to fit GEV, $H_{\gamma ,\mu ,\sigma }$, distribution to the block maxima, $M_{n,1},\ldots ,M_{n,m}$, that generates estimates for the parameters $\left(\hat{\gamma },\hat{\mu },\hat{\sigma }\right)$ is short $\hat{H}$ [30]. Note that GEV comprises the following three subclasses: for the parameter γ>0 named Fréchet (the distribution is heavy-tailed), for γ<0 and for γ=0 named Weibull. The theoretical return level when γ=0 for the GEV distribution indicates how the return level would look when the γ parameter equals zero, and all other coefficients remain constant.

The likelihood function for independent maxima $Z_{1},\ldots ,Z_{m}$, is given:

(2) $$\begin{array}{c} L\left(\gamma ,\mu ,\sigma \right)=-m\log\sigma -\left(1+\frac{1}{\gamma }\right)\sum_{i=1}^{m}\log\left[1+\gamma \left(\frac{Z_{i}-\mu }{\sigma }\right)\right]\\ -\sum_{i=1}^{m}{\left[1+\gamma \left(\frac{Z_{i}-\mu }{\sigma }\right)\right]}^{-1/\gamma } \end{array}$$

provided that 1+γ(z−μ)/σ>0, for i=1,…,m.

Coles and Dixon [31] proposed a penalty function for the maximum likelihood method for the GEV distribution. The penalty is in terms of both bias and variance magnitudes. The penalty function is of the form:

(3) $$P\left(\gamma \right)=\{\begin{array}{c} 1\;\;\mathrm{if}\;\gamma \leq 0\\ \exp\left\{-\lambda {\left(\frac{1}{1-\gamma }-1\right)}^{\beta }\right\}\;\mathrm{if}\;0<\gamma <1\\ 0\;\;\mathrm{if}\;\gamma \geq 1 \end{array}$$

for a range of non-negative values of λ and β. Therefore, the penalized likelihood function is as follows:

(4) $$L_{pen}\left(\gamma ,\mu ,\sigma \right)=L\left(\gamma ,\mu ,\sigma \right)\times P\left(\gamma \right)$$

Coles and Dixon [31] advise setting λ=β=1, which leads to reasonable performance. The Penalized Maximum Likelihood (PML) estimator appears to be slightly better than, or at least as good as, the PWM estimator. Following Coles and Dixon [31], the two approaches are applied for comparison, i.e., the penalized maximum likelihood (PML) and the maximum likelihood (ML) estimators.

The selection of the blocks corresponds to a period of a specific time length (e.g., one year). In the case of the block method, an alternative type of quantile is of interest. Generally, a return level $R_{n,T}$ with a return period T is the quantile of the maxima distribution $M_{n}$, which satisfies $P\left(M_{n}>R_{n,T}\right)=1/T$ [32]. The return level $R_{n,T}$ is expected to exceed on average once every 1/T given period (e.g., for annual data 1/p years) with probability p. The block in which the exceedance happens is referred to as the stress period. If one assumes that there are m blocks and each of length n, these m blocks follows the GEV distribution, then estimates of extreme quantiles $R_{n,T}$ of the maximum distribution are obtained by inverting the GEV distribution, as follows:

(5) $$R_{n,T}=\{\begin{array}{c} H_{\hat{\gamma },\hat{\mu },\hat{\sigma }}^{-1}\left(1-\frac{1}{T}\right)=\hat{\mu }-\frac{\hat{\sigma }}{\hat{\gamma }}\left(1-{\left(-log\left(1-\frac{1}{T}\right)\right)}^{-\hat{\gamma }}\right)\;\mathrm{for}\;\gamma \neq 0\\ H_{\hat{\gamma },\hat{\mu },\hat{\sigma }}^{-1}\left(1-\frac{1}{T}\right)=\hat{\mu }-\hat{\sigma }\left(-log\left(1-\frac{1}{T}\right)\right)\;\mathrm{for}\;\gamma =0 \end{array}$$

$H_{\hat{\gamma },\hat{\mu },\hat{\sigma }}^{-1}$ is the quantile function for GEV distribution (in short ${\hat{H}}^{-1}$).

Suppose that n=250, i.e., approximately 250 working days in a year and T=10, then $P\left(M_{250}>R_{250,10}\right)=1/10$. Then, one can infer that the return level is expected to be exceeded on average once every ten years. The variance of the return level $R_{n,T}$ is obtained by using the delta method [33]:

(6) $$Var\left(R_{n,T}\right)=Var\left(R_{p}\right)\approx \nabla R_{p}^{T}V\nabla R_{p}$$

where $\nabla R_{p}^{T}=\left[\frac{\partial R_{p}}{\partial \mu },\frac{\partial R_{p}}{\partial \sigma },\frac{\partial R_{p}}{\partial \gamma }\right]=\left[1,-\gamma^{-1}\left(1-y_{p}^{-\gamma }\right),\sigma \gamma^{-2}\left(1-y_{p}^{-\gamma }\right)-\sigma \gamma^{-1}y_{p}^{-\gamma }lny_{p}\right]$, $y_{p}=-\ln\left(1-p\right)$ and V is the variance-covariance matrix of $\left(\hat{\gamma },\hat{\mu },\hat{\sigma }\right)$.

The confidence interval for $R_{n,T}$ with a significance level α equals:

(7) $$P\left\{R_{n,T}-z_{\alpha }Var\left(R_{n,T}\right)<{\hat{R}}_{n,T}<R_{n,T}+z_{\alpha }Var\left(R_{n,T}\right)\right\}=1-\alpha$$

To assess the estimates, one can use a return level plot, which comprises a graph of $R_{n,T}=\mu -\frac{\sigma }{\gamma }\left(1-{\left(-log\left(1-1/T\right)\right)}^{-\gamma }\right)$ against $y_{p}=-\log\left(1-p\right)$ using a logarithmic scale.

Confidence intervals and empirical estimates can be added to the plot. If the GEV model is suitable for the data, then empirical and theoretical estimates should be the same. Generally, more accurate confidence intervals are obtained by evaluating the profile likelihood for the return level. The profile likelihood is a method for making inference on a particular parameter of $\theta_{i}$ of parameters vector θ. The log-likelihood for θ can be written as $l\left(\theta_{-i}\right)$, where $\theta_{-i}$ denotes all parameters of θ excluding $\theta_{i}$. The profile log-likelihood for $\theta_{i}$ is defined as $l_{p}\left(\theta_{i}\right)=\underset{\theta_{-i}}{\max}l\left(\theta_{-i}\right)$. The profile log-likelihood is the maximum log-likelihood with respect to all remaining parameters in θ excluding $\theta_{i}$.

In this approach, one parameter (e.g., $\gamma =\gamma_{0}$) is fixed, and the log-likelihood function is maximized with respect to the remaining parameters (μ,σ). This process is repeated for different values of $\gamma_{0}$.

To obtain confidence intervals for the return level, it is required to re-parameterize the GEV distribution so that $R_{n,T}$ is one of the parameters of the GEV. It is done by rearranging Equation (5) as follows:

(8) $$\hat{\mu }=R_{n,T}+\frac{\hat{\sigma }}{\hat{\gamma }}\left(1-{\left(-log\left(1-1/T\right)\right)}^{-\hat{\gamma }}\right)$$

and then replacing μ in Equation (1) with Equation (8). Consequently, it is possible to perform maximization for the return level as one of the parameters of the GEV distribution. The GEV parameter vector $\psi =\left(\gamma ,R_{n,T},\sigma \right)$ is partitioned into two components i.e., $\psi_{1}=R_{n,T}$ and $\psi_{2}=\left(\gamma ,\sigma \right)$, and the log-likelihood profile is defined as:

(9) $$L\left(\psi_{1}\right)=\underset{\psi_{2}}{max}\left(\psi_{1},\psi_{2}\right)$$

Under suitable regularity conditions for large samples, the deviance statistics is defined as:

(10) $$D\left(\psi_{1}\right)=2\left(L\left(\hat{\psi }\right)-L\left(\psi_{1}\right)\right)\sim \chi_{1}^{2}$$

The set of values $CL_{\alpha }$ for which $D\left(\psi_{1}\right)\leq CL_{\alpha }$ gives a (1−α) confidence interval for $R_{n,T}$, where $CL_{\alpha }$ is the (1−α) quantile of the $\chi_{1}^{2}$ distribution.

If the extremes show a tendency to form clusters (dependency), it is reasonable to inquire about their sizes. To do that, following [31], define the extremal index. Let 0≤θ≤1 and τ>0. Then, one can find a sequence $u_{n}\left(\tau \right)$ such that the following hold:

(11) $$\begin{array}{c} \underset{n\to \infty }{lim}n\left(1-F\left(u_{n}\left(\tau \right)\right)\right)\\ \underset{n\to \infty }{lim}P\left\{M_{n}\leq u_{n}\left(\tau \right)\right\}=exp\left(-\theta \tau \right) \end{array}$$

where $u_{n}$ is a non-decreasing sequence of real numbers, and F(·) denotes the marginal distribution. A straightforward way to estimate the extremal index θ is to use the blocks method. The asymptotic estimator of θ is:

(12) $${\hat{\theta }}_{BM}=n^{-1}\frac{log\left(1-K_{u}/m\right)}{log\left(1-N_{u}/mn\right)}$$

where $K_{u}$ is the number of blocks in which the threshold is exceeded, m is the number of blocks, $N_{u}$ is the number of exceedances over the threshold u and n is the number of observations in the m-th block. Smith and Weissman [34] investigate the statistical properties of such an estimator as the problem of choosing n (or m) and u. Note that both m and n should be large. Setting n=20 observations in the m-th block is deemed fine [34].

O’Brien [35] proposes the runs method where a high threshold u is used as well, but the estimator of θ is based on the runs, which means observations that are below the threshold u are utilized:

(13) $${\hat{\theta }}_{RM}=\frac{\sum_{i=1}^{n-r}I_{A_{i,n}}}{\sum_{i=1}^{n}I_{\left\{X_{i}>u\right\}}}=\frac{\sum_{i=1}^{n-r}I_{A_{i,n}}}{N_{u}}$$

where $A_{i,n}=\left\{X_{i}>u,X_{i+1}\leq u,\ldots ,X_{i+r}\leq u\right\}$ and r is the number of observations below the threshold. Computations by [34] suggest that the runs estimator has a lower bias than the blocks method.

A more recent proposal comes from [36]. They propose an interval estimator based on the rescaled inter-exceedance intervals. Suppose $N_{u}$ exceedances over a threshold value u and $1\leq t_{1}<\cdots <t_{N_{u}}\leq n$ is the time of these exceedances; then the proposed estimator takes the following form:

(14) $${\hat{\theta }}_{1}=\frac{2{\left(\sum_{i=1}^{N_{u}-1}T_{i}\right)}^{2}}{\left(N_{u}-1\right)\left(\sum_{i=1}^{N_{u}-1}T_{i}^{2}\right)}$$

where $T_{i}=t_{i+1}-t_{i}$ for $i=1,\ldots ,N_{u}-1$.

Ferro and Segers [36] propose another estimator of θ with the bias correction:

(15) $${\hat{\theta }}_{2}=\frac{2{\left(\sum_{i=1}^{N_{u}-1}\left(T_{i}-1\right)\right)}^{2}}{\left(N_{u}-1\right)\left(\sum_{i=1}^{N_{u}-1}\left(T_{i}-1\right)\left(T_{i}-2\right)\right)}$$

The estimator of θ ensures the estimate is within the region of 0 and 1 and is defined as:

(16) $${\hat{\theta }}_{FS}=\{\begin{array}{c} 1⋀{\hat{\theta }}_{1}\;\;if\;\;max\left\{T_{i}:1\leq i<N_{u}-1\right\}\leq 2\\ 1⋀{\hat{\theta }}_{2}\;\;if\;\;max\left\{T_{i}:1\leq i<N_{u}-1\right\}>2 \end{array}$$

The findings of Süveges [37] support the conjecture that the inter-exceedances estimator possesses the best characteristics. The mean cluster size of extreme values is obtained as reciprocal of θ.

## 3. EVT in Modeling and Forecasting of Delivery Delays

### 3.1. Data Characteristics

Using the tools described in Section 2, actual delivery data are analyzed. High-frequency data covering 3770 observations over approximately three months were taken into account.

It is crucial how the estimated time of arrival is calculated. The starting point is to set the actual time of the vehicle loading in the distribution center. Then, using a digital map, the distance and route are determined to the place of distribution included in the delivery order. Calculations take into account the restrictions related to the gross vehicle weight and road speed limitations. The average speed of travel on individual routes is determined based on history. This allows selecting the estimated time of arrival (ETA). In addition, due to the provisions of EC Regulation WE561, the rest time is set for routes that cannot be completed in the primary dimension of driving a vehicle by a driver, i.e., during 9 h of driving a day. If this happens, we will add 11 h of driver rest time to the ETA.

In the described delivery process, about 70–100 vehicles are engaged in the transportation, depending on the demand for transportation services. Thus, delays are not generated by one driver or one vehicle. They occur in different periods in real-time due to vehicle position registration and loading/unloading using GPS/GPRS technology (via intelligent transport system; ITS). The process is homogenous in that one transport company is offering its service to one supplier (a brewery company). The brewery product (beer) can be thought of as homogenous in transportation. It is packed and loaded in a standardized way. The number of receivers is defined as well. In a short period (three months), the receivers can be assumed constant (possible changes are irrelevant). Additionally, the routes are established in advance.

One can consider the causes of delays in transportation from three perspectives:

1. The management perspective, i.e., cargo management, determining optimal routes, analysis of the delivery time from the standpoint of planning, the technical and operational readiness of vehicles, and proper structure of contracts with subcontractors.
2. The road conditions perspective related to the possibility of dynamic changes on the road such as the occurrence of random events and hazards on the road (accidents, collisions), congestion, and changes in weather conditions.
3. The driver’s behavior perspective, including defective implementation of the plan, received from transport managers, psychophysical condition of the driver (exhaustion, stress), and irrational behavior on the road.

Delays appear stochastically, and they are treated as a realization of a stochastic process. Unless the transport management system remains unchanged, delays are time-dependent, although not assigned to an equally spaced time set.

**Figure 1:**
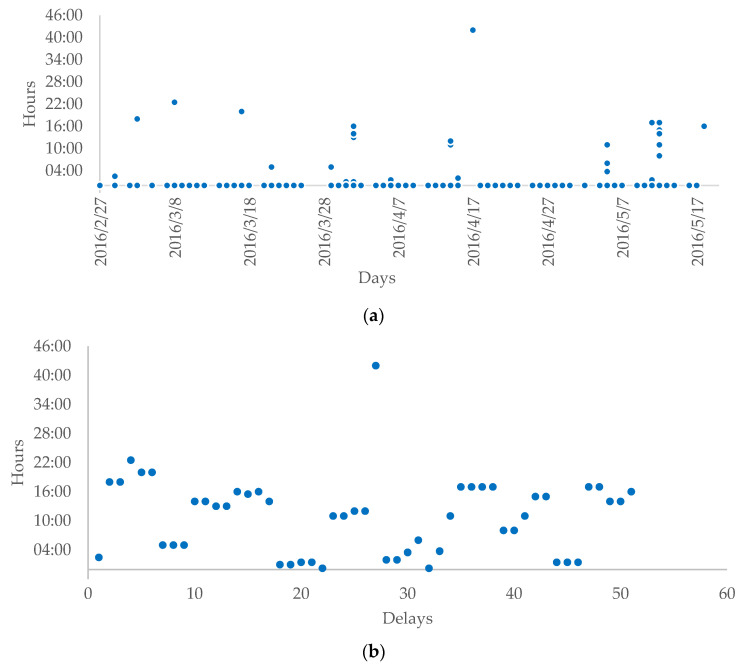
Delivery delays. Panel (**a**) covers all observations (3770 obs), and panel (**b**) covers delivery delays (51 obs).

The time series comprises the delays, i.e., the difference between the expected and the actual delivery time, expressed in hours (Figure 1). Due to many transport operations and a relatively small number of delays (51 delays in total), the ratio of delays equals 1.35% of the total.

Table 1 compared the characteristics of the entire set of the data (3770 observations) and delay data (51 observations). The minimum for all deliveries equals zero hours, i.e., delivery was before the expected time, so there is no delay.

The comparison illustrates that when the total data set is considered, delays are not significant. The median is equal to zero, and the mean value equals 8 min 48 s. However, standard deviation and shape parameters (skewness and kurtosis) indicate that the rare data distort the distribution. Looking at delay data (51 observations), it becomes clear that the observed delays are from 15 min to 42 h, which corresponds to the scale of possible consequences for a given carrier and the entire supply chain. The average delay is around 11 h (10:59:45), taking the mean, and 12 h taking the median. The standard deviation of almost 8 h shows considerable variability in delays, while shape parameters indicate departures from ideal values in Gaussian distribution. The latter is supported by the Jarque-Bera test [38] results, allowing rejecting the hull hypothesis assuming normality. Our findings are in line with [23]. They described time delays in travel time. State-of-the-art practices often use the mean and variance of travel time to derive buffer time and planning time indices as measures of road reliability. However, empirical travel time analysis showed that travel time distribution is not symmetrical but highly skewed to the right with a heavy tail, especially in the presence of road network disruptions resulting from adverse weather conditions, car collisions, or other incidents.

Figure 1 presents two plots with the delivery delays distributed over time. The upper one depicts all deliveries (namely 3770 observations) where deliveries with no delays are assigned zero hours. We can see that some of the delays happened on the same day, i.e., indicating some link between delays. The lower plot depicts only delays that are presented consecutively for better visibility. Most of the delays are less than 24 h, with one notable exception where the delay reached 42 h.

### 3.2. The Empirical Results

The analyzed type of time series data is called a randomly distributed zero-inflated time series and has specific properties [39,40,41]. The time series can be viewed as two processes when delays occur or not. In practice, we are interested in modeling the delays only, so the focus is directed at the maxima series rather than the expected value. That is why the time series consisting of maxima, i.e., delays in hours, are used. Then, the EVT is employed. The maximum likelihood (ML) method is applied to estimate the parameters of the GEV distribution. For small samples, the maximum likelihood estimator is biased, and its variance is higher than for the case of other methods, such as the Penalized Maximum Likelihood method (PML).

Table 2 shows estimates of the GEV distribution parameters with standard errors and *p*-values of significance for both cases, ML and PML. Only the parameter γ is insignificant (ML), while the others are highly significant. The maximized log-likelihood and the Bayesian Information Criterion (BIC) indicate the penalized ML method estimates’ advantage [42].

**Figure 2:**
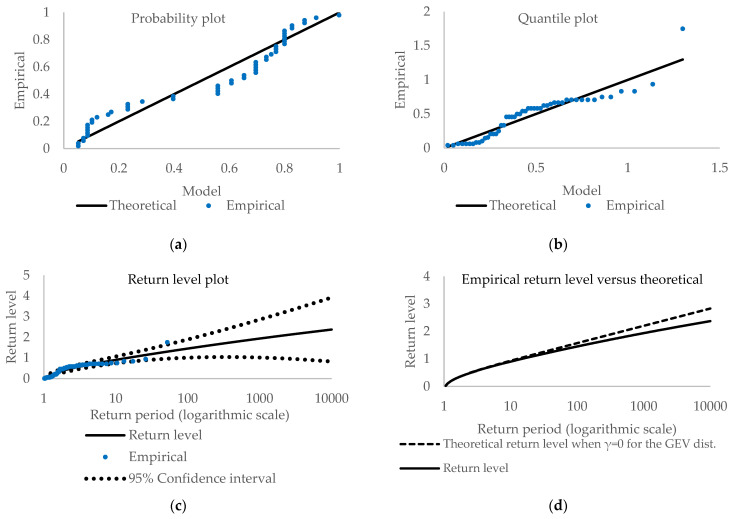
Diagnostic plots for Generalized Extreme Values distribution (ML method).

**Figure 3:**
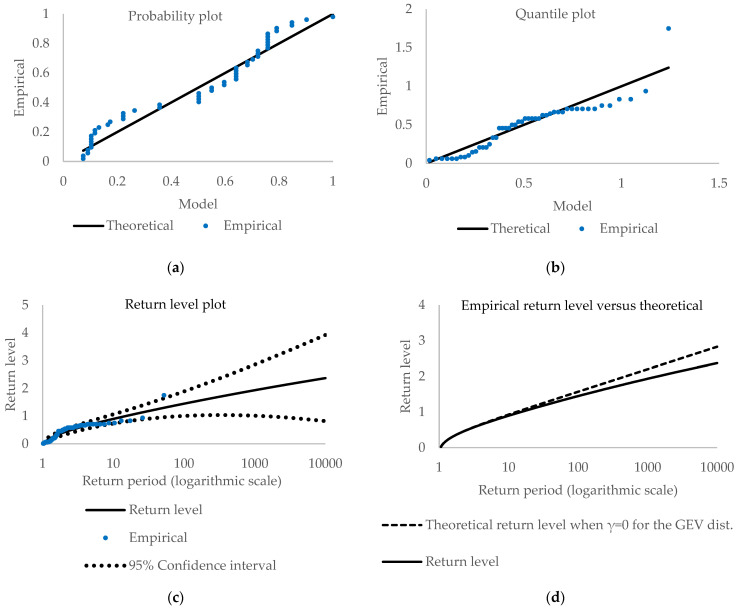
Diagnostic plots for Generalized Extreme Values distribution (PML method).

Figure 2 and Figure 3 depict various plots, i.e., panel (a)—probability, panel (b)—quantile, and panel (c) and (d) return level plots, as diagnostic tools (More about diagnostic plots and their interpretations can be found in [33,43,44,45].). For the probability plot and quantile plot, the points should lie close to the unit diagonal. Significant deviations from linearity mean that the model is not adequate for the data. The probability plot is the empirical distribution function evaluated at $z_{i}$, which are ordered extreme values. Consequently, the probability plot consists of the points $\left\{\left(\hat{H}(z_{i}\right),\;\left(i/\left(m+1\right)\right)\right\},\;i=1,\ldots ,m$ where m is the sample size. The quantile plot consists of the points $\left\{\left({\hat{H}}^{-1}\left(i/\left(m+1\right)\right)\right),\;z_{i}\right\},\;i=1,\ldots ,m$ where ${\hat{H}}^{-1}$ is the quantile function for GEV distribution line.

The plotted empirical points are close to linear, indicating that the GEV model is valid for extrapolation purposes, although there are some deviations from linearity, especially in the probability plot. The quantile plots are quite linear except for one observation. The variability of the observations likely causes the departures from linearity. Nonetheless, all plots seem to favor the PML over the ML methods. The probability and the quantile plots are similar. However, a small distinction exists. A significant difference is observed for the return level plots, where confidence intervals are narrower for penalized ML than for the ML method.

To check the validity of the observed plots, we employed the Anderson-Darling [46], Cramer [47] and von Mises [48] tests to check the goodness-of-fit of the fitted GEV distribution. The Anderson-Darling test is one of the most powerful tests based on the empirical distribution function. Table 3 presents results for both cases, i.e., the maximum likelihood method and the penalized maximum likelihood method. At 5% significance level, we do not reject the null hypothesis, so there is no evidence against assuming that the empirical distribution is from the GEV distribution for both estimation methods. However, the Anderson-Darling test at 10% significance level indicates rejection of the null so that it may be improved upon.

**Figure 4:**
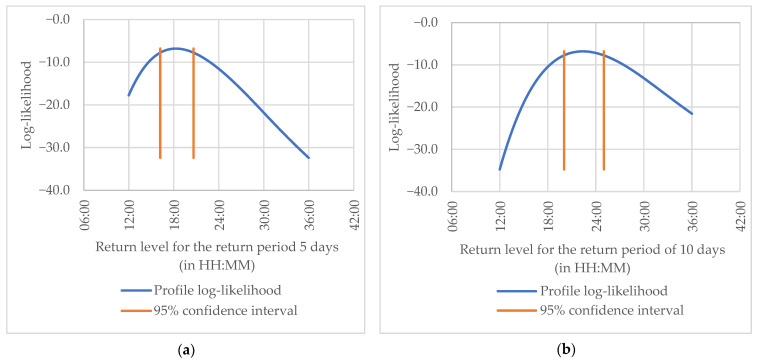
The profile likelihood for the return level when the return period is 5, panel (**a**), and 10 days, panel (**b**).

Figure 4 shows the profile log-likelihood estimated for the return level when the return period is 5 and 10 days from the PML. The 95% confidence intervals (orange lines) are rather symmetrical, and the bell-curve shape of the profile log-likelihood indicates that both estimates are fairly accurate.

**Figure 5:**
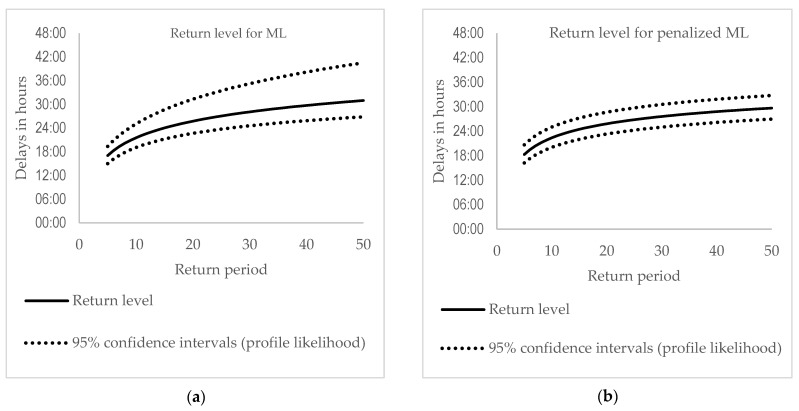
Return level estimates with 95% confidence intervals obtained from the profile likelihood for the ML method, panel (**a**) and the PML, panel (**b**).

Figure 5 displays a range of return levels and their confidence intervals obtained from the profile likelihood method. Firstly, it should be noted that Confidence Intervals (CIs) using the Penalized ML method are closer to the return levels than those obtained from the ML method. Secondly, the CIs for the PML are close to being symmetrical, contrary to the CIs from the ML method, where considerable asymmetry is visible. It indicates that estimates of the return levels and CIs from the PML are less uncertain in estimating the bands, meaning they have smaller differences between the upper and the lower bands than those obtained from the ML method. The PML estimates indicate that for every five delays, one expects to have a maximum delay of about 18 h and 16 min with 95% confidence intervals of (16:12; 20:38).

This indicates that estimates of the return levels and CIs from the PML are more appropriate than those obtained from the ML method. For every five delays, one expects to have a maximum delay of about 18 h and 16 min with 95% confidence intervals of (16:12; 20:38). For every ten delays, this is, respectively, about 22 h and 22 min with a 95% confidence interval of (20:02; 25:00). 

It is interesting to infer the implied cluster size of the series of extreme delays. It is reasonable to assume that one delay can cause another, which means that delays may form clusters. For the original time series (3770 observations), there are on average 60 deliveries per day, so setting 63 blocks gives nearly 60 observations per block. By contrast, the median of deliveries equals 66, generating 57 blocks. Setting the latter number of observations per block enables us to make interpretations in terms of days. It is common practice when it comes to the extremal index [30,32]. Table 4 shows the extremal index estimates using three different estimators, i.e., blocks method, runs method, and Ferro and Segers method.

The results show that delays tend to form clusters with a mean length between two and three days. Since the [36] estimator generates more accurate values than others, it is expected to be more trustworthy. These findings indicate that it is reasonably likely that one delay causes further delays.

## 4. Discussion

Divery is inevitably a part of the supply chain. Supply chain management is typically described as a proactive relationship and integration among various tiers in the chain [49]. Delivery represents a link between different elements and subcontractors [50,51,52,53].

The empirical results show that the extreme value theory applied to delivery delays is a promising tool of a supply chain distortion analysis. The presented approach is experience-based. It makes the assessment of delivery dependent on Key Performance Indicators [54]. One of them is timeliness, expressed by the ratio of ‘correct’ deliveries measured by the number of deliveries made on time to the total number of completed transports. The second is the delivery quality measured by the number of damaged cargo units to the total number of transported units in a certain number of transport operations. 

Since the EVT application in the present study is relatively novel in the specified area, we recommend including the EVT module in the integrated transport management system, being a part of the supply chain management system. It would be beneficial at the stage of anticipating delays and planning for reserves of vehicles and drivers. As the entire process is repeatable, finding optimal paths and time windows for deliveries within the framework of different constraints is possible. However, everyday practice indicates that rare but highly impactful delays are responsible for the distortion of the supply chain system, including optimizing the transportation processes.

Using the EVT as a tool of permanent monitoring and planning transportation in the supply chain requires a perfect information system integrated over all parties involved [55]. The optimal solution is using autonomous information collection without human intervention. Such systems are already utilized in just-in-time logistics, particularly in the production sector, e.g., automotive [54]. As concerns the theoretical model, it is straightforward that the delivery delays follow the process:

(17) $$y_{t}=\{\begin{array}{c} z_{t}\;\;\;\mathrm{if}\;\mathrm{there}\;\mathrm{is}\;a\;\mathrm{delay}\\ 0\;\;\;\;\;\;\mathrm{otherwise} \end{array}$$

It is a randomly distributed zero-inflated time series [39,40,41]. The time series can be viewed as two processes, whether delays occur or not. It is specific in the sense that we focus only on $z_{t}$ i.e., delays because zero is a normal state of no delays. Delays are distributed randomly. Thus, the process dynamics are different from the Ornstein-Uhlenbeck process, as was considered in [56]. It is often called a jump-diffusion process or the Ornstein–Uhlenbeck processes driven by the Lévy process [57,58]. Here, the state of diffusion is stable and equal to zero. The Process (20) can be modeled in different ways. One of the most often representations is the Markov model with Poisson distribution for count data [59].

Future works would include a comparison of the EVT model with the Markov zero-inflated Poisson model. The possible development is related to identifying factors of delivery delays as well. In addition, it would also be interesting to extend the analysis by studying the possibility that deliveries are the nonstationary extremes.

## 5. Conclusions

The purpose of this paper was to model and forecast rare events that appear as delivery delays in road transport using the Extreme Value Theory (EVT). We evaluated both the magnitude and frequency of delivery delays. The actual data set originated from the intelligent transport system (ITS) from a large European brewery company, and the cooperating road transport company has been studied. The results show that out of 3770 transport operations under study, only 51 delays have occurred, i.e., a 1.35% ratio, and the discrepancy between the plan and its implementation is low. This number justified applying the extreme value approach.

The empirical results positively answer the research questions. The extreme value theory is the appropriate tool for analyzing delivery delays since it gives estimated Generalized Extreme Value (GEV) distribution quantiles that fit the actual distribution. Estimating the extremal index in a considered process shows that the logistics operator should plan additional resources (vehicle and driver) every two to three days to guarantee a smooth delivery and flexible supply chain management.

The advantage of the EVT is that it allows considering any period. If the business environment is stable, the results of the EVT are stable as well. Its application can be easily extended for prediction, planning, and decision-making in any means of transportation as a part of a supply chain.

## Figures and Tables

**Table 1:** Descriptive statistics [HH:MM:SS].

No. of Obs.	Min	Max	Median	Mean	Std. dev.	Kurt	Skew	JB Stat	JB *p*-Value
3770	00:00:00	42:00:00	00:00:00	00:08:48	01:32:52	228.939	13.366	8,131,073	0.000
51	00:15:00	42:00:00	12:00:00	10:59:45	07:54:33	5.665	0.942	22.634	0.000

Note: The JB denotes the Jarque-Bera test for normality.

**Table 2:** Estimates of the Generalized Extreme Values (GEV) distribution.

**ML**	**Coefficient**	**Std. Error**	***p*-Value**
*γ*	−0.0452	0.0981	0.6449
*μ*	0.3129	0.0431	0.0000
*σ*	0.2731	0.0316	0.0000
Log likelihood	−12.7468	BIC	0.7312
**Penalized ML**	**Coefficient**	**Std. Error**	***p*-Value**
*γ*	−0.1802	0.0434	0.0000
*μ*	0.3435	0.0473	0.0000
*σ*	0.3182	0.0402	0.0000
Log likelihood	−6.8146	BIC	0.4985

**Table 3:** The goodness-of-fit tests.

	ML		PML	
	Statistic	*p*-Value	Statistic	*p*-Value
Anderson-Darling	1.8794	0.1071	2.0173	0.089
Cramer-von Mises	0.3096	0.1269	0.2923	0.1420

**Table 4:** Extremal index estimation results.

	Blocks Method	Runs Method	Ferro and Segers
Extremal index $\hat{\theta }$ (m=63)	0.4403	0.2941	0.3193
${\hat{\theta }}^{-1}$ (m=63)	2.27	3.40	3.13
Extremal index $\hat{\theta }$ (m=57)	0.3931	0.3137	0.3193
${\hat{\theta }}^{-1}$ (m=57)	2.54	3.19	3.13

Note: The assumed number of exceedances over a high threshold *N_u_* = 51.

## Data Availability

The data presented in this study are openly available in RepOD, V1 at https://doi.org/10.18150/FZS9EI (accessed on 21 June 2021).
